# Integrating Sama Dilaut culture: a case study for inclusion in the Philippine school improvement plan

**DOI:** 10.3389/fpsyg.2026.1785303

**Published:** 2026-08-05

**Authors:** Pattra Sakib Ugis, Nilo Jayoma Castulo, Zeda Sulanting-Sangkula, Kharsum H. Mohammad, Nadzmin Jalani Dugasan, Darwisa Sali-Ondo, Radzmal Soriano Abbani, Ajilma Majid Tambihasan, Alsadjaik Ytang Sabdul, Radzmalyn Abdurahman Akmad, Russel Bradecina Reyes, Solita Utoh-Asim Jemsy, Almira Amilbangsa-Hadji, Al-Sabrie Yusop Sahijuan, Shernaiza A. Hajad

**Affiliations:** 1 Department of Educational Leadership and Professional Services, College of Education, Mindanao State University–Tawi-Tawi College of Technology and Oceanography, Bongao, Tawi-Tawi, Philippines; 2 Department of Public Administration, College of Arts and Social Sciences, Mindanao State University–Tawi-Tawi College of Technology and Oceanography, Bongao, Tawi-Tawi, Philippines; 3 Department of Political Science, College of Arts and Social Sciences, Mindanao State University–Tawi-Tawi College of Technology and Oceanography, Bongao, Tawi-Tawi, Philippines

**Keywords:** cultural integration, inclusive education, Indigenous learners, Sama Dilaut (Badjao) culture, school improvement plan

## Abstract

This qualitative case study examined the integration of Sama Dilaut (Badjao) culture into the School Improvement Plan (SIP) to foster inclusive education in the Philippines. Framed by Vygotsky’s sociocultural theory and the Department of Education’s Indigenous Peoples Education (IPEd) framework, this study explored how cultural integration creates equitable learning environments for Indigenous learners. A qualitative case study design was employed. Data were collected through key informant interviews with eight educators from public schools in Bongao, Tawi-Tawi, who implemented culturally responsive SIP initiatives. The data were analyzed using thematic analysis. Thematic analysis identified five core strategies: (1) inclusive leadership practices, (2) cultural programming in schools, (3) cultural awareness and appreciation, (4) curriculum integration of Indigenous content, and (5) community and stakeholder partnerships. These strategies fostered a culturally grounded pedagogy, learner confidence, participation, and identity affirmation. However, persistent challenges, including poverty, early marriage, and language barriers, continue to hinder educational continuity. The findings suggest that embedding Badjao culture within SIP transforms schools into culturally meaningful environments that empower learners and strengthen community ties. The study recommends institutionalizing Indigenous pedagogy training, enhancing school-community collaboration, and developing policy frameworks to sustain cultural inclusivity. Ultimately, the research affirms that education becomes most transformative when aligned with learners’ cultural lifeworlds, promoting identity, equity, and social cohesion.

## Graphical abstract

**Figure:**
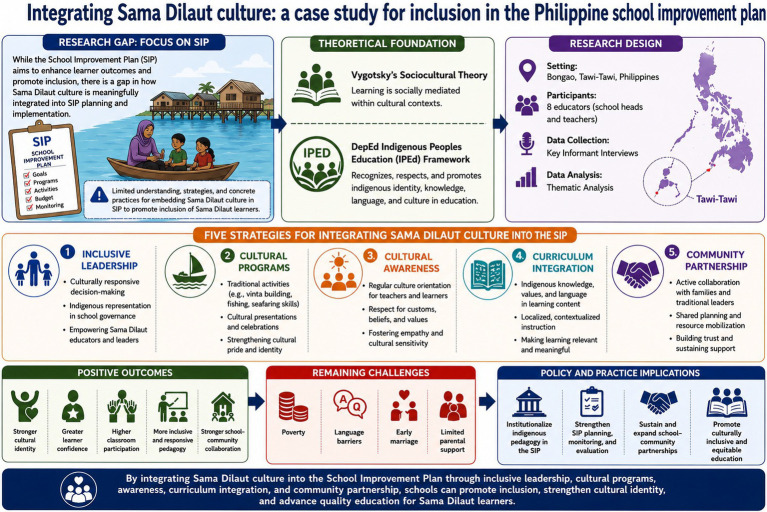

## 1 Introduction

Inclusion in education is a concept that focuses on incorporating all students into mainstream classrooms, irrespective of their personal, social, economic, or cultural backgrounds. It represents a dedication to establishing educational settings where diversity is recognized, respected, and appreciated. The goal of inclusion is to ensure equal educational opportunities for every student, including those with disabilities and those from minority or Indigenous communities (Engelbrecht et al., 2009; Hernández and Sardain, 2022; Honkasilta et al., 2024; Karim and Hue, 2022; Prater, 2009). Additionally, the significance of global practices in incorporating Indigenous cultures into education offers valuable perspectives for addressing the needs of the Sama Dilaut (Badjao) community in the Philippines. Schools incorporate Indigenous culture into their curricula through various methods designed to promote an inclusive and culturally responsive educational atmosphere. In New Zealand, Indigenous culture has been integrated into mathematics lessons by incorporating Indigenous languages and cultural contexts within the classroom. This method aids students in comprehending complex identities and supports a culturally sustaining pedagogy (Meaney et al., 2013). In Australia, teachers participate in participatory action research (PAR) cycles to gain a deeper understanding of Indigenous content and its incorporation into their teaching practices. This approach aligns their teaching with social justice and equity objectives (Baynes, 2016). Similarly, research in Canada has shown that while there is a willingness to integrate Indigenous knowledge, practical implementation encounters obstacles. Suggestions include enhancing collaboration with Indigenous communities and improving resource allocation (Kanu, 2005). However, challenges such as resistance to integrating Indigenous knowledge, insufficient resources, and the necessity for teacher training remain significant barriers (Kanu, 2005; Marshman and Strohfeldt, 2023; Michie et al., 2023).

Among the nation’s most marginalized Indigenous communities, the Sama Dilaut (Badjao) encounter ongoing educational obstacles stemming from social exclusion, cultural misrepresentation, and economic difficulties (Gonzaga and Pratama, 2025; Pahut et al., 2026; Sulanting-Sangkula et al., 2026). Research focusing on Indigenous learners with disabilities from the Alangan-Mangyan communities has brought to light issues related to equity, accessibility, and the cultural dimensions of education. These challenges are often intensified by the intersection of indigeneity and disability, indicating that Sama Dilaut (Badjao) learners may face similar difficulties (Salva, 2025). Therefore, fostering an organizational culture that emphasizes collaboration and cultural inclusivity can greatly improve educational quality. For instance, incorporating the Gija language and culture into the Boornoolooloo School’s curriculum has created a culturally responsive learning environment, leading to better educational outcomes (Mung et al., 2025). Additionally, integrating traditional values like Pikukuh from the Baduy community into conflict resolution education has been effective in enhancing students’ understanding of conflict resolution, promoting peace, and encouraging cooperative behavior (Puryanto, 2025). In the Philippines, the Department of Education (DepEd) has incorporated cultural diversity into the curriculum, especially in areas with significant Indigenous populations, such as Mindanao. This initiative includes the integration of Islamic values and basic Arabic grammar in predominantly Muslim regions and the accreditation of Indigenous People’s (IP) schools (Arquiza, 2006).

Nevertheless, these initiatives are characterized by a national perspective and an integrationist approach, rather than fully acknowledging and empowering traditional educational systems and Indigenous educational practices (Arquiza, 2006). Moreover, the Basic Education Reform Agenda (BESRA) and the conversion of the Non-Formal Education Bureau into the Bureau of Alternative Learning Systems (BALS) are intended to promote cultural integration within the educational framework. However, the execution of these plans is inconsistent, and significant progress toward fully recognizing Indigenous education systems is still absent (Arquiza, 2006). Additionally, the Mother Tongue-Based Multilingual Education (MTB-MLE) initiative seeks to improve learner inclusion and accessibility by incorporating native languages in early education. In schools where researchers studied Badjao (Sama-Dilaut) learners, Sinama, the language spoken by the Badjaos (Sama-Dilaut), is used as the medium of instruction in the primary grades to enhance comprehension and engagement. Nonetheless, there is an ongoing discussion about returning to a Filipino–English bilingual education model, which could affect the success of MTB-MLE and, consequently, the cultural inclusion of Indigenous students (Bravo-Sotelo et al., 2024). Despite policies that advocate for inclusive education, practical obstacles such as funding availability, social stigma, teacher training, and intergovernmental coordination continue to exist (Custodio, 2025). These challenges impede the complete achievement of inclusive education, likely impacting the cultural inclusion of Sama Dilaut (Badjao) learners. In addressing structural and policy challenges, the School Improvement Plan (SIP) strategies have encouraged genuine community empowerment. The program extends beyond cultural symbols by involving the Sama Dilaut (Badjao) community in decision-making, incorporating Indigenous knowledge, and engaging parents, elders, and local officials as educational partners. This involvement enables them to contribute to shaping educational priorities. Thus, this study aimed to examine the integration of Sama Dilaut (Badjao) culture into the SIP to foster inclusive education in the Philippines. This study seeks to answer the following research questions:

1. How do school leaders and teachers integrate Sama Dilaut (Badjao) culture into the implementation of the school’s improvement plan?
2. What are the influences of cultural inclusion on the educational performance of Sama Dilaut (Badjao) learners?

## 2 Literature review

### 2.1 Strategies for integrating Indigenous cultural identities in educational institutions

Educational institutions employ various strategies to integrate Indigenous cultural identities, such as Sama Dilaut (Badjao) culture, into their curricula and pedagogical practices to promote inclusivity in education. These strategies aim to preserve cultural heritage, enhance students’ cultural identities, and foster inclusive learning environments. Building on the digital preservation of culture, integrating Indigenous knowledge into the curriculum is a critical strategy for enhancing cultural inclusivity in education. For instance, utilizing digital tools to integrate Indigenous cultural heritage into educational practices, as seen in the Sami museum and archive sector, helps preserve and promote cultural heritage (Linkola-Aikio and Keskitalo, 2025). Additionally, incorporating Indigenous knowledge into the curriculum helps bridge traditional and modern education, enhancing cultural competency and social inclusion (Chen and Shih, 2025; Cruz, 2025). For example, the Sistema Educativo Indígena Propio (SEIP) in the Andean region integrates Indigenous and external knowledge, reflecting community worldviews (Cruz, 2025). This integration also extends to professional development by infusing cultural heritage into teacher education programs that influence teaching identity and classroom management, thereby promoting culturally relevant curricula (Gadaza et al., 2025). Similarly, in the Philippines, higher-education institutions have integrated local cultural content into classroom instruction using storytelling and community-based projects to affirm cultural identities and promote intercultural competence (Sales-Batang et al., 2025). These educational institutions play a vital role in promoting the integration of Indigenous cultural identities by adopting inclusive and culturally responsive teaching practices. These efforts not only preserve cultural heritage but also empower marginalized communities and enhance students’ sense of belonging and cultural identity.

### 2.2 Influences of Indigenous cultural practices on marginalized learners

The inclusion of Indigenous cultural practices in multicultural learning environments significantly impacts the academic performance, engagement, and sense of belonging of marginalized learners, such as the Sama Dilaut (Badjao). Engaging with learners’ cultural backgrounds and lived experiences as classroom resources increases their engagement. This approach has been effective in Aboriginal education, where it harnesses students’ backgrounds and interests, leading to improved educational outcomes (Burgess and Evans, 2016, 2019). In addition, creating quality learning environments that mobilize family and community social and cultural capital helps marginalized students feel a sense of belonging and receive sociocultural support (Burgess and Evans, 2016). Indigenous cultural development support does not directly impact GPA; however, it indirectly influences it through an enhanced sense of belonging and perception of institutional support (Fong et al., 2021). Additionally, integrating Indigenous cultural practices strengthens students’ cultural identity and boosts their confidence and sense of agency, fostering a stronger sense of belonging within their schools and communities (Pu et al., 2024). In the context of Philippine education, using local and Indigenous literary texts enhances student engagement by reflecting on their own languages and customs and fostering a deeper emotional connection to the material (Castro-Caliboso et al., 2025). Thus, incorporating Indigenous cultural practices into education not only enhances academic performance and engagement but also fosters a strong sense of belonging among marginalized learners. Educators can support the comprehensive development of these students by creating inclusive and culturally responsive learning environments that ultimately lead to better educational outcomes and greater social equity.

### 2.3 Integration of Indigenous cultures into school improvement plan

Community participation and stakeholder collaboration are vital for integrating Indigenous cultures, such as Sama Dilaut (Badjao), into school improvement plans. Indigenous perspectives on community participation highlight the importance of trust-based relationships and reciprocal learning. These elements are essential for effective participatory processes in Indigenous primary healthcare services and can be applied in educational settings (Turner et al., 2019). Additionally, programs such as ‘Young Doctors for Life’ demonstrate the importance of adapting initiatives to ensure cultural relevance. Successful adaptation requires engaging local communities to align their programs with their cultural contexts and goals (Good et al., 2024). This approach ensures that programs are meaningful and effective for Indigenous children and their families. In addition, collaboration between public officials and Native American communities has positively impacted organizational performance and substantive student outcomes. However, it has less impact on process-oriented outcomes such as joint problem-solving and cross-cultural learning (Conner, 2017). This suggests that while collaboration improves direct outcomes, more effort is needed to enhance collaborative processes. These approaches collectively support the integration of Indigenous cultures, such as the Sama Dilaut (Badjao), into educational frameworks, promoting cultural competency and inclusivity and improving educational outcomes.

### 2.4 Theoretical underpinning

This study is grounded in Vygotsky’s Sociocultural Theory, which emphasizes that learning is a socially mediated process shaped by culture, language, and interaction within the learner’s environment. Vygotsky posited that cognitive development does not occur in isolation but through participation in culturally meaningful activities facilitated by knowledgeable others, such as teachers, peers, parents, and community members. Within this framework, education is understood as a collaborative endeavor in which cultural tools and social contexts shape how learners construct knowledge, identity, and competence. In the context of this study, the integration of Sama Dilaut (Badjao) culture into the School Improvement Plan (SIP) aligns with Vygotsky’s idea of creating a *“social situation of development,”* where learners’ growth is supported by culturally relevant experiences and social interactions. By embedding local traditions, language, and community values into the learning process, schools provide Sama Dilaut (Badjao) learners with environments that resonate with their lived realities and cultural identities. This congruence between learners’ sociocultural backgrounds and school environments fosters meaningful engagement, motivation, and cognitive development. The Department of Education’s (DepEd) Indigenous Peoples Education (IPEd) Framework operationalizes these theoretical principles within the Philippine educational context. The framework promotes education that is culture-based, responsive, and community participatory, ensuring that learning processes reflect Indigenous worldviews and ways of knowing (Department of Education, 2011). This provides a policy foundation for schools to integrate Indigenous knowledge systems, languages, and practices into curriculum design and school governance, thus enacting the sociocultural principle that learning is most effective when it builds on learners’ existing cultural foundations. The strategies identified in this study, such as curriculum integration of Indigenous culture, community engagement and partnerships, and culturally responsive leadership, illustrate how Vygotsky’s theory manifests in practice through the IPEd framework. For instance, integrating Sama Dilaut (Badjao) stories and language into classroom instruction serves as a cultural tool that mediates learning and connects abstract concepts to familiar experiences. Similarly, partnerships with parents, elders, and barangay officials exemplify guided participation, in which community members act as co-educators who scaffold learners’ understanding in their cultural context. Through these culturally congruent learning environments, schools effectively construct a social development situation that empowers Sama Dilaut (Badjao) learners to achieve academic success. Within this context, education is not merely an academic pursuit but a process of cultural affirmation and identity formation. By aligning pedagogy, policy, and community engagement with learners’ sociocultural realities, schools can transform into spaces where Indigenous learners can thrive, enhancing their academic performance, self-esteem, cultural pride, and social participation. Thus, the integration of Vygotsky’s SCT and the DepEd Indigenous People Education (IPEd) Framework provides a theoretical and operational foundation for understanding how culturally responsive education can bridge the gap between traditional knowledge and formal schooling. This synergy ensures that the inclusion of Sama Dilaut (Badjao) culture within the SIP does not simply adapt Indigenous learners to the school system but rather transforms the school into a culturally meaningful environment that supports their holistic development and well-being of the students.

## 3 Methodology

### 3.1 Research design

A review of the related literature revealed several gaps in existing studies on Indigenous education and cultural inclusion in the Philippines’ educational system. While previous research has highlighted global models for integrating Indigenous knowledge into school curricula, such as in New Zealand, Australia, and Canada, few studies have examined how these approaches are localized within the Philippine educational framework, particularly through the School Improvement Plan (SIP). Moreover, although the Department of Education’s Indigenous Peoples Education (IPEd) framework advocates for culturally responsive and community-based learning, there is insufficient empirical evidence on its actual implementation in marginalized Indigenous communities, such as the Sama Dilaut (Badjao). Prior studies have also underscored the role of leadership, community partnerships, and culturally responsive pedagogy in promoting inclusion; however, few have explored how these elements interact in real-world school contexts. These gaps highlight the need for context-specific research that captures the lived experiences and practices of schools serving Indigenous learners.

In this study, ‘educational performance’ is conceptualized qualitatively to encompass learners’ perceived engagement in classroom activities, confidence in participating in academic and cultural school events, and sense of identity affirmation within the learning environment. Rather than measuring standardized test scores or grade point averages, this study examines how cultural inclusion influences learners’ motivation, self-expression, and belonging—factors that educators identified as critical indicators of meaningful educational participation.

To address these limitations, the present study adopted a qualitative case study design to explore and describe the integration of Sama Dilaut (Badjao) culture into the school improvement plan for the school. This approach enables a deep understanding of how inclusive leadership, community engagement, and cultural integration are operationalized in actual school settings. The case study design is particularly suited for examining complex social phenomena within their real-life contexts, allowing researchers to capture the nuanced experiences of school leaders and teachers who implemented the SIP for Sama Dilaut (Badjao) learners. In addition, this study considers the perspectives of Sama Dilaut (Badjao) community members, such as parents, elders, and local leaders, to ensure that their voices, cultural values, and lived experiences help illustrate how the SIP was designed and implemented. Through this design, the study not only documents existing practices but also provides insights into how culturally grounded education can be strengthened to promote inclusivity and equity in the classroom.

### 3.2 Research locale and participants

This study was conducted in selected public schools in Bongao, Tawi-Tawi, Philippines, where a significant population of Sama Dilaut (Badjao) learners is enrolled in public schools. This area was purposefully chosen because it represents one of the most active educational settings implementing inclusive and culturally responsive programs using the School Improvement Plan (SIP). The locale provides an appropriate context for examining how schools operationalize cultural inclusion within an Indigenous community that has historically faced educational marginalization.

The participants were purposefully selected to ensure that information-rich cases were included in this study. Eight educators participated in the study, comprising three school principals, one school head, three class advisers, and one subject teacher. All had experience implementing SIP initiatives that integrated Sama Dilaut (Badjao) cultural elements into their programs. Their teaching and leadership experience ranged from seven to 22 years, ensuring diverse perspectives across different school levels (see Table 1). Each participant played a direct role in integrating cultural content into classroom instruction or school programs, making their insights crucial for understanding inclusive educational practices. In addition, the researchers use “Sama Dilaut (Badjao)” to align with official Department of Education records and the School Improvement Plan where the study was conducted. While “Sama Dilaut” is the community’s preferred endonym and “Badjao” may carry derogatory connotations, this paper’s usage is strictly contextual and not intended to disrespect the community’s identity.

**Table 1:** Demographic profiling.

Pseudonym	Gender	Position	Plantilla position	Years of teaching experience/managing position	Indigenous community affiliation	Integrate/implement Sama Dilaut culture into classroom teaching/SIP
Adamus	Male	School head	EGT-I	13 years	Sama	Yes
Deia	Female	Principal	ESP-I	7 years	Sama	Yes
Flamara	Female	Principal	ESP-I	17 years	Sama	Yes
Terra	Female	Class adviser	EGT-I	15 years	Bisaya	Yes
Pirena	Female	Principal	ESP-I	10 years	Sama	Yes
Mitena	Female	Class adviser	EGT-III	13 years	Sama	Yes
Olgana	Female	Class adviser	EGT-I	16 years	Sama	Yes
Zaur	Male	Subject teacher	EGT-II	22 years	Sama	Yes

SIP, EGT, and ESP stand for school improvement plan, elementary grade teacher, and elementary school principal, respectively. Plantilla positions are permanent, officially approved jobs by government offices or public institutions with a fixed salary grade and item number.

Prior to data collection, the participants were informed of the objectives of the study and provided informed consent. Participation was voluntary, and strict measures were taken to maintain confidentiality and anonymity. To ensure credibility and ethical integrity, pseudonyms were assigned to all participants, and identifying information was omitted from the data presentation.

### 3.3 Data collection and analysis

Data were collected through key informant interviews (KIIs) with selected school heads (principals), class advisers and subject teachers. This method was chosen because it allowed the researcher to obtain in-depth insights from individuals with direct experience implementing the school improvement plan (SIP) and integrating Sama Dilaut (Badjao) culture into school programs. The interview process aimed to explore the participants’ perceptions, experiences, and practices in promoting cultural inclusion and supporting the educational performance of Sama Dilaut (Badjao) learners.

Before conducting the interviews, the researcher secured the necessary permissions from the school authorities and obtained informed consent from each participant. The interviews were conducted in a convenient and comfortable setting for the respondents to ensure open and respectful dialogue. Each session lasted approximately 30–45 min and was audio-recorded with the participants’ consent to ensure the accuracy of the data. Field notes were also taken to capture nonverbal cues and contextual observations. Ethical considerations were strictly observed; participation was voluntary, responses were kept confidential, and participants could withdraw from the study at any time without any consequences.

After data collection, all recordings were transcribed verbatim and reviewed for accuracy. The transcripts were read, coded, and grouped into categories to form themes based on the research questions. Frequency counts were used to identify the most prevalent themes. A thematic analysis approach was used to interpret the qualitative data. This process involved several stages: data cleaning, initial coding, categorization of codes into themes and interpretation. Thematic patterns were derived inductively, allowing the findings to emerge naturally from the participants’ responses. The codes were grouped into broader categories that reflected recurring ideas and experiences related to leadership practices, cultural integration, community partnerships, and learner outcomes. Additionally, the coded data were reviewed using ATLAS.ti version 26 to organize patterns, check the consistency of themes, and strengthen the analysis. This ATLAS.ti version 26 served only as a support tool, while all interpretations and final theme validation were handled by the researchers to ensure transparency and academic integrity of the study. To ensure the trustworthiness of the findings, the researcher engaged in member checking by allowing participants to review and validate the interpretation of their statements. Triangulation was achieved by comparing data across different participants and schools to enhance the credibility and reliability of results.

## 4 Results

This study presents the strategies employed by schools to integrate Sama Dilaut (Badjao) culture into their school improvement plan (SIP) and the corresponding influence of cultural inclusion on learners’ educational performance. Through careful thematic analysis of the interview data, several major themes emerged, reflecting how school leaders and teachers operationalized inclusion in their educational practices.

Summarizes the themes, codes, and frequencies that emerged from the qualitative analysis (Table 2). These themes illustrate a comprehensive view of how inclusion is enacted, from leadership and curriculum design to community partnerships and learner empowerment. Each theme represents a distinct but interrelated dimension of cultural integration, highlighting the collective efforts of educators and stakeholders to promote inclusive, culturally grounded learning environments for Sama Dilaut (Badjao) students.

**Table 2:** Summary of themes.

Category	Themes	Top codes	Frequency
Strategies for integrating Sama Dilaut (Badjao) culture into the school improvement plan.	1. Leadership practice for inclusive education	Teachers’ and students’ management Community partnership and engagement Cultural sensitivity in leadership Cultural inclusion in SIP Conflict resolution and discipline	22
	2. Highlighting the Indigenous culture in school programs	Cultural performances and arts Traditional attire and expression Student participation and motivation Parental involvement	14
	3. Cultural awareness and appreciation	Recognition of diverse cultural identities Respect for Sama Dilaut (Badjao) customs and traditions Integration of Indigenous values in lessons	56
	4. Curriculum integration of Indigenous culture	Infusion of cultural content in subjects Inclusion of arts, dances, and local crafts Language-based cultural lessons	46
	5. Community engagement and partnership	Parents’ collaboration in school projects Barangay officials’ active participation	45
Influences of cultural inclusion on Sama Dilaut (Badjao) Learners’ educational performances	1. Challenges in cultural integration	Early marriage practices Lack of academic motivation Limited parental support Language Barriers	13
	2. Positive impacts of cultural inclusion on learners	Improved self-confidence through cultural exposure Enhanced participation in school activities Development of social skills and cooperation Recognition of identity and belongingness	16
	3. Inclusive pedagogy and teaching strategies	Differentiated instruction for Sama Dilaut (Badjao) learners Use of culturally relevant materials Flexible learning standards and assessments	49
	4. Learner empowerment and participation	Increased self-confidence among Sama Dilaut (Badjao) learners Active involvement in school cultural events Improved classroom participation	53

The authors have created this table.

The results are presented in two main categories aligned with the research questions.

1. Strategies for Integrating Sama Dilaut (Badjao) Culture into the School Improvement Plan, and
2. Influence of Cultural Inclusion on Sama Dilaut (Badjao) Learners’ Educational Performance.

These themes are further elaborated in the following sections, supported by direct excerpts from the participants’ narratives, to illustrate their lived experiences and perspectives.

### 4.1 Strategies for integrating Sama Dilaut (Badjao) culture into the school improvement plan

The analysis revealed five major themes representing how schools integrate Sama Dilaut (Badjao) culture into their school improvement plans (SIPs): (1) Leadership practices for inclusive education, (2) Highlighting Indigenous Culture in School Programs, (3) Cultural Awareness and Appreciation, (4) Curriculum Integration of Indigenous Culture, and (5) Community and stakeholder partnerships. These themes reflect the multifaceted efforts of educators and administrators to promote cultural inclusion through leadership, pedagogy and collaboration.

#### 4.1.1 Leadership practice for inclusive education

Inclusive education begins with leadership that listens to, understands, and values the cultural backgrounds of all the learners. The findings showed that school heads and teachers who demonstrate cultural sensitivity and inclusive leadership are instrumental in fostering an environment in which Sama Dilaut (Badjao) learners feel acknowledged and respected. Their leadership extends beyond administrative functions, embodying empathy and community engagement. By actively involving parents and Sama Dilaut (Badjao) elders in school initiatives, leaders gain a deeper understanding of learners’ cultural contexts and challenges, allowing them to design relevant and responsive programs for the students. They shared that:

*“Cultural sensitivity in leadership is essential for creating an inclusive atmosphere.”*

*“Leaders must engage with the community to understand the unique needs of Sama Dilaut (Badjao) learners.”*

*“Conflict resolution strategies should reflect the cultural context of the Sama Dilaut (Badjao) community.”*

Participants emphasized the importance of culturally sensitive leadership, stating, for instance, that ‘Cultural sensitivity in leadership is essential for creating an inclusive atmosphere’ and that “Leaders must engage with the community to understand the unique needs of Sama Dilaut (Badjao) learners,” highlighting the need for context-specific approaches, even in conflict resolution. Inclusive leadership is demonstrated when school heads involve community elders in planning activities and decision-making, which strengthens the school’s commitment to equity and diversity.

#### 4.1.2 Highlighting Indigenous culture in school programs

Schools that celebrate the heritage and identity of their learners create a more engaging and inclusive atmosphere. Participants described how cultural events, such as Buwan ng Wika and Indigenous Peoples Education (IPEd) Month, serve as platforms to showcase Sama Dilaut (Badjao) dances, songs, attire, and crafts. These school-wide activities enhance students’ sense of pride and belonging, while promoting cultural visibility and mutual respect among learners from different backgrounds. They shared that:

*“Cultural performances and arts can significantly boost student participation.”*

*“Traditional attire and expression should be celebrated in school events.”*

*“Parental involvement is key to reinforcing cultural values in education.”*

Such programs also encourage collaboration between schools and communities, reinforcing the notion that cultural inclusion is a shared responsibility of educators, families and local stakeholders. In practice, these activities may include cultural presentations. Schools often integrate these events into their regular school calendars so that cultural recognition becomes a continuous practice rather than a one-time celebration.

#### 4.1.3 Cultural awareness and appreciation

Promoting awareness and appreciation of diverse cultural identities is a central element in fostering inclusivity. Teachers who integrate Indigenous values and customs into classroom discussions cultivate empathy, respect, and understanding among their students. Participants emphasized that when Sama Dilaut (Badjao) learners see their culture represented in lessons, they feel respected and valued, which enhances their motivation to learn. They shared that:

*“Respect for Sama Dilaut (Badjao) customs and traditions is vital for student identity.”*

*“Integration of Indigenous values in lessons promotes cultural appreciation.”*

*“Recognition of diverse cultural identities enhances the learning experience.”*

This theme highlights that education is not merely about knowledge acquisition, but also about nurturing intercultural understanding and mutual respect, which are the essential foundations of inclusive schooling. In classroom practice, this may involve discussing cultural traditions during lessons and activities.

#### 4.1.4 Curriculum integration of Indigenous culture

Integrating Sama Dilaut (Badjao) cultural elements directly into the curriculum makes learning more contextual and meaningful. Teachers incorporate local crafts, traditional arts, stories, and language into various subjects to connect academic content with learners’ lived experiences to this end. This approach bridges the gap between home and school while reinforcing the cultural identities of learners. They shared that:

*“Infusion of cultural content in subjects enriches the educational experience.”*

*“Inclusion of arts, dances, and local crafts fosters cultural pride.”*

*“Language-based cultural lessons help bridge communication gaps.”*

Such a curriculum promotes both academic engagement and cultural sustainability, ensuring that Indigenous knowledge is preserved while learners gain formal education. In practical terms, curriculum integration may involve using traditional stories in language lessons, exploring geometric concepts through Indigenous craft patterns, or discussing environmental stewardship based on local ecological knowledge.

#### 4.1.5 Community engagement and partnership

The study found that the successful integration of culture in schools depends greatly on active partnerships with parents, the elderly, and Brgy. officials. Their participation in planning, implementing, and monitoring school programs ensures that cultural inclusion remains authentic and relevant to the community’s needs. These partnerships build trust, cooperation, and shared ownership, which are vital for the long-term sustainability of inclusive educational initiatives. They shared that:

*“Parents’ collaboration in school projects enhances cultural relevance.”*

*“Barangay officials’ active participation is crucial for community support.”*

*“Building partnerships with stakeholders fosters a sense of ownership in education.”*

This theme reinforces the interconnectedness of education and culture; effective inclusion is sustained when community members view schools as partners in cultural preservation and learner development. Community engagement in practice may include regular consultation meetings, the involvement of elders as cultural resource persons, and joint monitoring of school initiatives.

### 4.2 Influences of cultural inclusion on Sama Dilaut (Badjao) learners’ educational performances

The second major category explores how cultural inclusion influences the educational experiences and performance of Sama Dilaut (Badjao) learners. The findings revealed that while inclusion fosters confidence, participation, and a stronger sense of identity, persistent challenges such as poverty, early marriage, and language barriers continue to negatively affect learners’ academic continuity. Five themes emerged from the analysis: (1) Challenges in cultural integration, (2) Positive impacts of cultural inclusion on learners, (3) Inclusive pedagogy and teaching strategies, (4) Learner empowerment and participation, and (5) Community support and engagement.

#### 4.2.1 Challenges in cultural integration

Despite schools’ efforts to integrate cultural elements into education, the participants identified several barriers that limited full inclusion. Common challenges include early marriage, economic hardship, limited parental support, and language barriers, which hinder academic motivation and attendance. These issues reflect the socio-cultural realities of the Sama Dilaut (Badjao) community, where traditional norms and survival often take precedence over formal education in their lives. They shared that:

*“Early marriage practices often limit educational opportunities for Sama Dilaut (Badjao) girls.”*

*“Language barriers create significant challenges in academic performance.”*

*“Limited parental support can negatively impact student motivation.”*

For instance, for “early marriage,” explain how it specifically limits educational opportunities for girls in this community (e.g., cultural expectations, economic pressures, and lack of alternatives). Furthermore, these challenges highlight the need for culturally responsive interventions that extend beyond the classroom, such as community awareness programs, parental engagement, and government support, to ensure educational continuity and inclusion.

#### 4.2.2 Positive impacts of cultural inclusion on learners

When their culture is acknowledged and valued in school, Sama Dilaut (Badjao) learners demonstrate notable improvements in their confidence, participation, and academic engagement. Teachers observed that culturally inclusive programs led to greater self-esteem and a willingness to participate in academic and extracurricular activities. This sense of recognition fosters belonging and motivation, transforming education into an empowering experience. Participants noted that:

*“Improved self-confidence through cultural exposure leads to better academic outcomes.”*

*“Enhanced participation in school activities reflects a sense of belonging.”*

*“Development of social skills and cooperation is evident among culturally included learners.”*

This perspective affirms that cultural validation strengthens learners’ identities and motivates them to engage more actively in their educational journeys.

#### 4.2.3 Inclusive pedagogy and teaching strategies

Teachers’ ability to adapt their instruction plays a key role in supporting culturally diverse learners. The findings revealed that educators employed differentiated instruction, visual learning aids, and culturally relevant materials to make lessons more accessible to Sama Dilaut (Badjao) students. They also modified assessments and classroom management strategies to accommodate the linguistic and cultural diversity. They shared that:

*“Differentiated instruction for Sama Dilaut (Badjao) learners ensures equitable access to education.”*

*“Use of culturally relevant materials enhances engagement and understanding.”*

*“Flexible learning standards and assessments accommodate diverse learning styles.”*

These practices reflect a shift from standardized instruction toward inclusive pedagogy, which values each learner’s background and adjusts teaching to promote equitable learning opportunities for all the students.

#### 4.2.4 Learner empowerment and participation

Cultural inclusion directly impacts learners’ sense of agency and their participation. When Sama Dilaut (Badjao) students are encouraged to share their traditions and lead cultural activities, they develop leadership skills, self-expression, and a strong sense of identity. Participants described how inclusion transformed students from passive recipients to active contributors to school life. They shared that:

*“Increased self-confidence among Sama Dilaut (Badjao) learners is evident in their classroom participation.”*

*“Active involvement in school cultural events promotes a sense of pride.”*

*“Improved classroom participation reflects the positive impact of cultural inclusion.”*

This indicates that learner empowerment arises when schools create spaces for representation, voice, and cultural expression, helping learners take pride in their heritage while excelling academically.

## 5 Discussion

The integration of Sama Dilaut (Badjao) culture into the School Improvement Plan (SIP) reveals a comprehensive and systematic approach to inclusive education that underscores the dynamic relationship between culture, leadership, and student development. While interpreting these findings, we acknowledge that the perspectives presented here are derived exclusively from school-based personnel, which risks institutional or “top-down” bias, potentially overstating success or overlooking community-specific tensions regarding cultural integration that might be voiced by Sama Dilaut (Badjao) learners and elders.

The findings affirm that culturally responsive leadership and pedagogy play vital roles in ensuring that education becomes relevant, empowering, and equitable for marginalized learners. Consistent with global research by Baynes (2016), Kanu (2005), and Meaney et al. (2013), this study demonstrates that inclusive leadership practices are the cornerstone of cultural integration. School heads and teachers who practice cultural sensitivity and engage directly with the Sama Dilaut (Badjao) community can create environments where diversity is celebrated rather than merely accommodated. Their participatory approach strengthens trust and collaboration between schools and communities, which are key factors identified by Arguelles and Sarsale (2025) for improving educational outcomes in rural Philippine schools.

The findings also show that school-based cultural programs, such as traditional dances, songs, and language celebrations, enhance learners’ pride and participation in school. These activities go beyond symbolic recognition and function as vehicles of cultural affirmation and social cohesion. By giving visibility to Indigenous culture, schools promote a deeper sense of belonging and identity among Sama Dilaut (Badjao) learners, aligning with studies that highlight the role of culturally grounded education in boosting motivation and engagement (Castro-Caliboso et al., 2025; Pu et al., 2024). Moreover, curriculum integration of Indigenous content bridges the gap between academic knowledge and lived experience. Although some activities were only performed on special events and not fully included in the daily lessons, some teachers viewed cultural activities as symbolic rather than as inclusive and meaningful. Lessons that include local crafts, stories, and language contextualize learning and validate the cultural heritage of learners. This supports Akinsola (2025) view that culturally responsive education enhances students’ cognitive and affective outcomes by connecting learning to sociocultural realities. Importantly, this study emphasizes that community engagement and partnerships sustain inclusive education. Parents, elders, and local officials play active roles in designing and implementing school programs to ensure that cultural inclusion remains authentic and community-driven. Such collaboration aligns with the participatory models proposed by Turner et al. (2019) and Good et al. (2024), in which community engagement fosters mutual learning and collective accountability.

Despite these positive outcomes, persistent challenges such as early marriage, poverty, and language barriers remain significant obstacles. Some teachers resisted cultural integration because they lacked training on how to align Indigenous content with the curriculum. In some cases, integrating Indigenous content depended on individual teachers, leading to inconsistencies. These findings resonate with Custodio (2025) observation that gaps in resource allocation, teacher preparation, and policy implementation hinder the realization of inclusion in the Philippines. This shows that while SIP supports cultural inclusion, turning it into consistent classroom practices remains a challenge. Addressing these barriers requires coordinated interventions across the education, social welfare and local governance sectors. Although cultural programs increase pride and participation, issues such as early marriage and poverty limit school-based learning interventions. Ultimately, this study reinforces that inclusion is not a one-dimensional policy goal but rather a transformative process grounded in empathy, respect, and shared responsibilities. Cultural inclusion enhances not only learners’ academic performance, which is mainly understood through qualities such as classroom participation, confidence, and engagement in learning, but also their self-worth and sense of belonging.

### 5.1 Theoretical implications

The findings of this study provide significant insights into the theoretical understanding of inclusive and culturally responsive education, particularly in the context of Indigenous learners such as the Sama Dilaut (Badjao). The integration of Sama Dilaut (Badjao) culture into the School Improvement Plan (SIP) offers concrete evidence of how inclusion theories can be applied and adapted within localized, community-based educational systems. First, it reinforces culturally responsive pedagogy (CRP) as a guiding framework. CRP emphasizes the importance of connecting instruction to learners’ cultural backgrounds, validating their experiences, and using culture as a learning foundation. The findings demonstrate how teachers who incorporate Indigenous practices, such as storytelling, crafts, and local languages, make learning more meaningful and relevant for Sama Dilaut (Badjao) learners. This supports the theoretical view that culture is not peripheral to learning but is central to learners’ engagement and cognitive development (Burgess and Evans, 2016). Second, the results substantiate the co-participation (CoP) framework, which posits that learning occurs through social participation and shared meaning-making. Collaborative efforts between school leaders, teachers, parents, and community elders reflect how inclusive education thrives when schools function as communities of practice. This aligns with the study’s observation that cultural inclusion is most effective when educators and stakeholders engage in continuous dialogue and collective decision making. Third, the study’s outcomes support the social inclusion theory, which focuses on reducing barriers to participation and ensuring equitable access to resources and opportunities.

By integrating cultural content into the SIP, schools can create learning environments that acknowledge and affirm Indigenous learners’ social and cultural identities. This leads to improved self-esteem, participation, and academic outcomes, illustrating how inclusion fosters educational equity and social justice. Additionally, the findings extend the Department of Education’s Indigenous Peoples Education (IPEd) framework, which promotes culturally grounded and community-responsive education. This study operationalizes the framework by demonstrating how leadership, pedagogy, and community engagement interact to sustain inclusive practices at the school level. This shows that the DepEd IPEd framework principles can be effectively localized to address the unique needs of the Sama Dilaut (Badjao), bridging policy and practice. In summary, this research enriches existing theoretical perspectives by grounding them in the lived realities of Indigenous learners. This affirms that inclusion is most powerful when it is culturally embedded, community-driven, and contextually adaptive. By merging global theories of inclusion with local cultural frameworks, this study advances a model of education that not only teaches but also transforms and empowers learners while preserving their cultural identities.

### 5.2 Implications for policy and practice

The findings underscore the importance of translating inclusive education theories into actionable and context-specific policies. To ensure that cultural integration within schools, particularly among Indigenous communities such as the Sama Dilaut (Badjao), becomes both sustainable and transformative, several policy and practice recommendations have been proposed at the institutional, community, and national levels.

#### 5.2.1 Institutionalize cultural sensitivity and Indigenous pedagogy training

Teacher education institutions might consider a mandatory certification programs by integrating into pre-service curricula. Teacher education programs and in-service professional development should include comprehensive modules on culturally responsive teaching, Indigenous knowledge systems, and inclusive classroom management. This training will equip educators to recognize cultural diversity as an educational strength, rather than a challenge. Institutionalizing such capacity-building efforts aligns with the theoretical emphasis on culturally responsive pedagogy and ensures that teachers possess the competence and empathy to teach in multicultural environments.

#### 5.2.2 Strengthen community-based educational policies

Education policies should mandate school-community partnerships as integral components of the school improvement plan. Including Sama Dilaut (Badjao) elders, parents, and local leaders in the planning, implementation, and evaluation of school programs ensures that initiatives remain culturally grounded and community-owned. Policies that institutionalize this participatory approach would reinforce the community of practice model, promoting shared responsibility for learners’ holistic development.

#### 5.2.3 Address socio-cultural and economic barriers

For early marriage, this could involve collaborations with social welfare agencies for counseling and economic support programs for families. Programs such as conditional financial assistance, family literacy initiatives, and culturally adapted counseling services should be strengthened to support Indigenous learners’ educational continuity in the future. These measures align with social inclusion theory by ensuring equitable access to educational opportunities and social participation.

#### 5.2.4 Integrate Indigenous content across curriculum areas

Teachers should be encouraged and supported to incorporate Sama Dilaut (Badjao) stories, crafts, local languages, and traditional values across subjects. In doing so, it enhances academic engagement and preserves cultural identity within the learning process. Curriculum developers should design learning materials that reflect Indigenous perspectives to ensure that cultural inclusion is an integral aspect of education.

#### 5.2.5 Establish monitoring and evaluation frameworks for inclusion

Policymakers and education administrators might consider to implement systematic evaluation tools to monitor the outcomes of inclusion programs. Indicators such as learner attendance, participation, and academic performance should be regularly assessed to measure the effectiveness of cultural integration. The evaluation results can inform data-driven decision-making and continuous improvement within schools, consistent with the Department of Education’s Indigenous Peoples Education (IPEd) Framework.

#### 5.2.6 Promote intersectoral and intercultural collaboration

Inclusive education for Indigenous learners requires collaboration across multiple sectors, such as education, culture, social services, and local governance. Partnerships with non-government organizations, cultural institutions, and community leaders can amplify resources, strengthen program implementation, and ensure that policies reflect the lived realities of marginalized groups. Such collaborations transform inclusion from a school-level initiative into a collective social movement toward equity and justice.

Essentially, these policy and practice recommendations emphasize that inclusion must move beyond compliance toward genuine cultural empowerment. Schools that embrace Indigenous Knowledge Systems (IKS) not only improve learning outcomes but also uphold education as a right that affirms identity, dignity, and social cohesion.

### 5.3 Limitations and future directions

While this study provides valuable insights into the integration of Sama Dilaut (Badjao) culture into the School Improvement Plan (SIP), certain limitations must be acknowledged to contextualize its findings and to guide future research. First, the study’s scope and sample size were limited to selected schools in Bongao, Tawi-Tawi, which may not fully represent the diverse experiences of Sama Dilaut (Badjao) communities across different regions of the Philippines. Educational practices and cultural dynamics can vary depending on local governance, resource availability, and community engagement, suggesting that future studies should expand their geographical coverage to include a broader range of contexts. Second, the study used a qualitative case study design based primarily on interviews. While this approach enabled an in-depth understanding of participants’ lived experiences, it may also have been influenced by subjectivity, both from the participants’ interpretations and the researcher’s analytical perspective. To strengthen the validity and generalizability of future findings, researchers can adopt mixed-method approaches that combine qualitative insights with quantitative data on learner performance, attendance, and participation. Third, this study focused primarily on the perspectives of school leaders and teachers, which may inadvertently reflect institutional rather than community-centered views of inclusion—a significant limitation given the historical marginalization of the Badjao.

Future research could extend this inquiry by incorporating the voices of Sama Dilaut (Badjao) learners, parents, and community elders, thereby capturing a holistic understanding of cultural inclusion and its intergenerational impact on the community. Including learner-centered data would offer deeper insights into how inclusion shapes academic motivation, cultural identity, and social relationships. Finally, future studies may explore the long-term outcomes of integrating Indigenous culture into SIPs, including their effects on educational attainment, community cohesion, and cultural preservation. Such longitudinal research could serve as a valuable reference for policymakers and educators when designing sustainable inclusion programs. Although this study’s findings provide meaningful contributions to inclusive and culturally responsive education, addressing these limitations in future research will allow for a more comprehensive and empirically grounded understanding of how cultural integration can transform learning experiences for Indigenous communities such as the Sama Dilaut (Badjao) in the Philippines.

## 6 Conclusion

Integrating Sama Dilaut (Badjao) culture into the School Improvement Plan embodies an effort toward inclusive education that connects learning to learners’ social and cultural realities. Grounded in Vygotsky’s sociocultural theory, the findings suggest that education becomes more meaningful when Indigenous knowledge and experience are recognized and valued. The identified strategies–inclusive leadership, cultural programs, cultural awareness, curriculum integration, and community partnerships–function as interconnected mechanisms that support culturally responsive learning environments. These approaches demonstrate the potential for enhancing participation and a sense of belonging among Indigenous learners. Through this framework, SIP functions not only as an administrative plan but also as a social situation of development, where knowledge, identity, and participation intersect. Leadership practices serve as scaffolds for inclusion, school-based cultural programs, and curriculum integration operate as cultural tools that shape understanding, and community partnerships provide a broader social context that sustains development. According to the DepEd Indigenous Peoples Education (IPEd) framework, this theoretical model demonstrates that inclusive education is a pedagogical and sociocultural process. This affirms that meaningful learning emerges when schools acknowledge and integrate Indigenous knowledge systems, fostering both empowerment and equity. For Sama Dilaut (Badjao) learners, cultural integration within the SIP enhances academic engagement and performance and nurtures self-worth, pride, and a strong sense of belonging. Ultimately, the theory of culturally mediated inclusion posits that the success of inclusion lies in aligning education with learners’ cultural world. When leadership, pedagogy, and community converge within a shared cultural framework, education transcends its traditional function; it becomes a means of preserving identity, strengthening the community, and empowering generations toward social transformation. However, the study also reveals factors such as early marriage and poverty, indicating that while school-based initiatives contribute to inclusion, they cannot fully address structural barriers on their own. Therefore, meaningful progress requires sustained collaboration between schools, communities, and local institutions. Rather than claiming complete transformation, the findings highlight the value of gradual and context-sensitive efforts that respect cultural diversity while addressing its limitations. This balanced perspective reinforces that inclusion is an ongoing process that requires continuous reflection and improvement.

## Data Availability

The raw data supporting the conclusions of this article will be made available by the authors, without undue reservation.
